# The Utility of Preoperative Vascular Grading in Patients Undergoing Surgery First for Pancreatic Cancer: Does Radiologic Arterial or Venous Involvement Predict Pathologic Margin Status?

**DOI:** 10.1155/2018/7675262

**Published:** 2018-08-13

**Authors:** Neha Goel, Jimson W. D'Souza, Karen J. Ruth, Barton Milestone, Andreas Karachristos, Rajeswari Nagarathinam, Harry Cooper, John Hoffman, Sanjay Reddy

**Affiliations:** ^1^Department of Surgical Oncology, Fox Chase Cancer Center, 333 Cottman Avenue, Philadelphia, PA 19111, USA; ^2^Department of Biostatistics, Fox Chase Cancer Center, 333 Cottman Avenue, Philadelphia, PA 19111, USA; ^3^Department of Radiology, Fox Chase Cancer Center, 333 Cottman Avenue, Philadelphia, PA 19111, USA; ^4^Department of Pathology, Fox Chase Cancer Center, 333 Cottman Avenue, Philadelphia, PA 19111, USA

## Abstract

Controversy exists on accurately grading vascular involvement on preoperative imaging for pancreatic ductal adenocarcinoma. We reviewed the association between preoperative imaging and margin status in 137 patients. Radiologists graded venous involvement based on the Ishikawa classification system and arterial involvement based on preoperative imaging. For patients with both classifications recorded, we categorized vascular involvement as “None,” “Arterial only,” “Venous only,” or “Both” and examined the association of vascular involvement and pathologic margin status. Of 134 patients with Ishikawa classifications, 63%, 17%, 11%, and 9% were graded as I, II, III, and IV, respectively. Of 96 patients with arterial staging, 74%, 16%, and 10% were categorized as stages i, ii, and iii, respectively. Of 93 patients with both stagings, 61% had no vascular involvement, 7% had arterial only, 14% had venous only, and 17% had both involved. Ishikawa classification was strongly associated with a positive SMA and SMV margin (p<0.001). However, for arterial staging, there was no association with SMA or SMV margin. Overall, Ishikawa grading was more predicative of arterial involvement and remained significant on multivariate analysis. The use of diagnostic imaging in predicting positive margins is more accurate when using a venous grading system.

## 1. Introduction

Pancreatic ductal adenocarcinoma (PDAC) is one of the leading causes of cancer related mortality worldwide with a 5-year survival rate less than 20% [1]. While surgical resection remains the only curative option, more than 80% of patients present with unresectable disease [2, 3]. Historically, resectability of PDAC was defined by absence of distant metastases, absence of local tumor extension to the celiac axis (CA) and hepatic artery (HA), and lack of involvement of visceral vasculature. However, data from the 1990s suggested that vein resection with negative margins was associated with equivalent survival to standard pancreatoduodenectomy (PD), leading to an increasing acceptance of vascular resection for curative resections [4]. There is now emerging evidence that a subset of patients, categorized as borderline resectable (BR), may benefit from resection after neoadjuvant therapy due to the theoretical benefits of downstaging the tumor, treating subclinical micrometastatic disease, increasing the proportion of patients who will receive and complete therapy, and the ability to better monitor the tumor's response to therapy [5, 6]. Accurate radiographic imaging is therefore critical in the management of PDAC as it helps define resectability status which in turn helps with preoperative treatment planning.

To improve the rate of complete resection of the primary tumor to microscopically negative margins, expert consensus groups have developed criteria to define tumor resectability. The National Comprehensive Cancer Network (NCCN) currently defines a tumor in the head/uncinate process as BR if there is solid tumor contact with the superior mesenteric vein (SMV) or portal vein (PV) of greater than 180 degrees, contact of less than or equal to 180 degrees with contour irregularity of the vein or thrombosis of the vein but with suitable vessel proximal and distal to the site of involvement allowing for safe and complete resection and vein reconstruction. Solid tumor contact with the inferior vena cava (IVC) is also considered BR. In terms of arterial criteria, solid tumor contact with the common hepatic artery (CHA) without extension to the CA or HA bifurcation allowing for safe and complete resection and reconstruction and/or solid tumor contact with the superior mesenteric artery (SMA) less than or equal to 180 degrees is considered BR. In terms of a pancreatic body/tail solid tumor, contact with the CA of less than or equal to 180 degrees or solid tumor contact with the CA greater than 180 degrees without involvement of the aorta and with an intact and uninvolved gastroduodenal artery (GDA) is considered BR [7]. The MD Anderson Cancer Center (MDACC) defines BR as short-segment occlusion of the SMV, PV, or SMV/PV confluence with a normal SMV and normal PV above and below tumor involvement allowing for vascular reconstruction [8]. In terms of arterial involvement, abutment of the SMA, abutment or short-segment encasement of the HA, or abutment of the CA is considered BR. According to the Alliance criteria, tumor-vessel interface of greater than or equal to 180 degrees of the SMV or PV wall circumference and/or a reconstructable occlusion are considered BR. In terms of arterial involvement, tumor-vessel interface of less than 180 degrees of the SMA wall circumference, reconstructable short-segment interface of any degree between the tumor and the HA, and tumor-vessel interface of less than 180 degrees of the CA are considered BR [8].

More specific criteria looking at the type of vascular deformity also exist. In particular, the Ishikawa classification strictly looks at the angiographic venous deformity and is based on radiographic findings that demonstrate the relationship of the tumor to the SMV-PV as (1) normal; (2) a smooth shift without narrowing; (3) unilateral narrowing; (4) bilateral narrowing; and (5) bilateral narrowing and the presence of collateral veins. The goal of a more detailed classification system is to see if one can predict an R0 margin status since synchronous vascular resection can only be justified if a margin negative resection is achieved without increased morbidity and mortality, and if there is no invasion of the vessel wall [9]. Our group previously proposed the use of Ishikawa classification type II and III vein deformity as venous involvement criteria for BR tumors and has shown that these patients benefit from preoperative therapy [10]. The aim of this study is to examine the association of preoperative venous and arterial involvement on imaging to assess whether either or both can predict positive vascular margins in patients who had initial resection for PDAC.

## 2. Methods

A retrospective review of medical records approved by our institutional review board was performed. A total 137 patients who underwent surgery for resectable or BR PDAC at Fox Chase Cancer Center from 1993 to 2013 were included. Mesenteric vascular involvement was identified by clinical and operative notes, as well as preoperative abdomen computed tomography (CT) and portal phases of mesenteric angiogram reports, if available. We defined PV-SMV involvement by Ishikawa classification as (I) normal, (II) smooth shift without narrowing, (III) unilateral narrowing, (IV) bilateral narrowing, and (V) bilateral narrowing with collateral veins. Arterial involvement was classified by our group as (i) clean, (ii) dirty fat, and (iii) abutment. Furthermore, overall vascular involvement was classified as (Ishikawa I and arterial i) none, (I and ii/iii) arterial only, (II/III/IV and i) venous only, or (II/III/IV and ii/iii) both. All imaging was read by board certified radiologists with experience in abdominal-oncology. Pathology and operative reports were reviewed to determine margin status, tumor size, and details of the surgical resection. The specimen was marked at the operating table with sutures placed at the bile duct margin and the proximal and distal SMV and SMA margins. Marking clips were placed on the sutures to denote proximal and distal. The entire SMA margin was painted with red ink and the entire SMV margin with blue ink. Sections were then taken perpendicular to the entire SMV and SMA margins. This has been our standard orientation for approximated 20 years, initially initiated by the senior pancreatic surgeon. Resection margins were considered positive according to the definition proposed by American Joint Committee on Cancer 7th edition. Postoperatively, patients were followed up with history and physical examination, CA-19-9 levels and CT scans of the chest, abdomen, and pelvis. Follow-up data was extracted through June 2015.

Statistical analysis was performed using SPSS software (SPSS Inc., Chicago, IL). For univariate analysis, the chi-square test was used for categorical variables and either Mann–Whitney *U* or unpaired Student's *t*-test was used for continuous variables. Differences were considered statistically significant when* P *< 0.05. Overall survival (OS) rate was calculated as the time from the date of radiologic or histologic diagnosis to the date of death using Kaplan-Meier method and log-rank test was used to evaluate statistically significant differences. The association of vascular involvement and pathologic margin status (R0 at SMA, PV/SMV, or bile duct/pancreatic duct margins) was additionally examined using logistic regression.

## 3. Results

### 3.1. Patient Demographics

A total of 137 patients were included in this study (Table 1). The median age at the time of presentation was 70 years (range 38-97) and 55% were female. All patients underwent surgery first. Eighty-five percent had a PD without vascular resection and 13% had concomitant vascular resection (8% SMV, 0.7% SMA, 2.9% PV, and 1.5% HA resection). One hundred and nine patients (80%) received postoperative CRT and 24 (17.5%) had surgery without adjuvant therapy. Positive lymph nodes were identified in 100 (73%) patients. The baseline carbohydrate antigen (CA) 19-9 levels before surgery were recorded as normal-mildly elevated (< 50 U/ml) in 39 (28.5%), moderately elevated (50-250 U/ml) in 41 (29.9%), and severely elevated (> 250 U/ml) in 56 (40.9%) patients.

### 3.2. Clinicopathological Features by Ishikawa and Arterial Classifications

**Figure 1:**
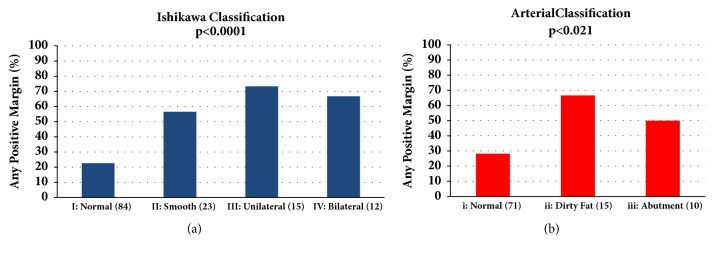
Margin status by (a) Ishikawa and (b) Arterial classification.

Of the 137 patients included in the study, 134 were staged according to the Ishikawa classification. Eighty-four patients (61%) were Ishikawa type I (no venous involvement), 38 (28%) were Ishikawa types II and III (unilateral involvement), and 12 (9%) were Ishikawa type IV (bilateral narrowing). None of the patients were Ishikawa type V. Three patients had unknown venous staging. Ninety-six patients were staged by degree of arterial involvement. Seventy-one (52%) were stage i, 15 (11%) were stage ii, and 10 (7%) were stage iii. Overall, both Ishikawa and arterial classifications showed significant differences between no vascular involvement and vascular involvement in the context of positive margins (P < 0.0001) (Table 2). Among patients with unilateral venous involvement (Ishikawa types II and III), 24 (63%) were positive for any margins. In particular, when looking at positive vascular margins, 18 (47%) had a positive SMA margin and 11 (29%) had a positive SMV margin. Among patients with bilateral venous involvement (Ishikawa type IV), 8 (67%) were positive for any margins (Figure 1(a)). Seven (58%) had a positive SMA margin and 4 (33%) had a positive SMV margin (p < 0.0001) and (p = 0.0001), respectively, when compared to Ishikawa type I (Table 2(a)). These findings suggest that Ishikawa staging was strongly associated with positive SMA and SMV margins. However, when categorized based on the degree of arterial involvement, despite significant differences between the presence of arterial involvement and positive margin status (p < 0.0001) (Figure 1(b)), the degree of arterial involvement showed no association with positive SMA (P = 0.062) or SMV margins (P = 0.63) (Table 2(b)).

**Figure 2:**
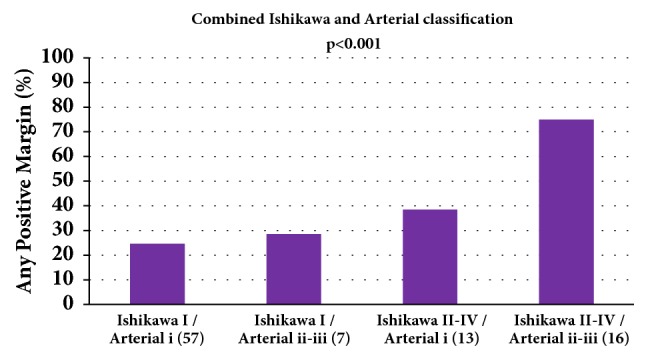
Combined Ishikawa and Arterial classifications and margin status.

When looking at preoperative imaging for the 93 patients in whom combined preoperative arterial and venous staging was performed, 57 (61%) patients had no vascular involvement (Ishikawa I and arterial i), 7 (8%) had arterial involvement only (I and ii/iii), 13 (14%) had venous involvement only (II/III/IV and i), and 16 (17%) had both arterial and venous involvement (II/III/IV and ii/iii). When comparing positive margin status and vascular involvement, Ishikawa staging had a higher probability of predicting any positive margins for both isolated venous (39%) and combined arterial and venous (75%) involvement (P < 0.0001) (Figure 2).

On multivariate analysis both Ishikawa and arterial grading systems were able to significantly predict any positive margins (p = 0.0023 and 0.0046, respectively) (Table 3). However, only Ishikawa staging significantly predicted both positive SMA (p = 0.0036) and SMV (p = 0.048) margins. While arterial grading was able to predict positive SMA margins (p = 0.020), it did not predict positive SMV margins (p = 0.68). Combined venous and arterial grading was significant at predicting both SMA (p = 0.0243) and SMV (p = 0.0084) margins.

### 3.3. Additional Factors Associated with Positive Margins

We further examined whether lymph node involvement and CA 19-9 levels are associated with any positive vascular margins. On univariate analysis, positive lymph node status was associated with a positive venous margin by Ishikawa staging (OR = 2.5, p = 0.04). There is no correlation between lymph node status and SMA margin by arterial staging (OR = 2.7, p = 0.55). On multivariate analysis, adjusting for lymph node status and CA 19-9 levels, combined venous and arterial staging was significantly associated with any positive (p = 0.0083) and positive SMA (p = 0.03) margins (Table 4).

### 3.4. Survival Analysis

**Figure 3:**
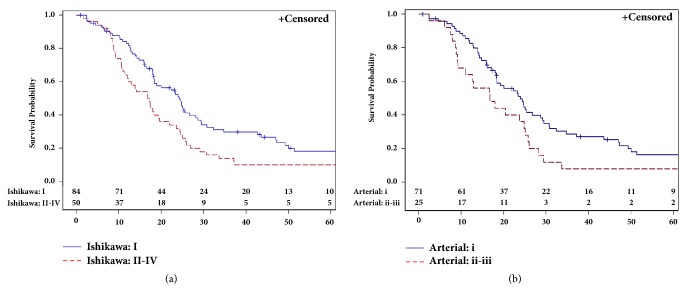
Overall survival by (a) Ishikawa and (b) arterial classification.

Kaplan-Meier OS curves are shown in Figure 3. Estimated median OS for the entire study cohort was 19.6 (95% CI = 17.9 – 24.4) months. For patients who underwent Ishikawa staging the estimated 3-year OS was 29.8% in patients without venous involvement and 14% in patients with venous involvement (p = 0.0056). The 3-year OS rate for patients who underwent arterial staging was 28.7% in patients without arterial involvement and 8% in patients with arterial involvement (p = 0.0422). When staged by combined venous and arterial involvement, there was no significant difference in 3-year OS between patients with and without vascular involvement. The 3-year OS was 28.7% for neither venous nor arterial involvement, 20% for either venous or arterial involvement, and 12.5% for both venous and arterial involvement (p = 0.2981) (Table 5). On univariate analysis, adjuvant therapy (p < 0.0001) and CA 19-9 levels (p = 0.022) were independently associated with OS.

## 4. Discussion

Optimal treatment planning for patients with BR resectable PDAC is challenging. A critical point in the decision-making process is to determine if a tumor can be resected with negative margins, as that is the best chance for cure [9]. This study examines the association of both venous and arterial involvement on preoperative imaging to assess whether either can predict positive pathologic margins. By preoperatively identifying those who may have an R1 vascular margin, we can better identify those patients who may benefit from neoadjuvant therapy.

Historically, resectability of pancreatic cancer was defined by absence of distant metastases, absence of local tumor extension to the CA and HA, and lack of involvement of the superior mesenteric vasculature. However, data from the 1990s suggested that vein resection with negative margins was associated with equivalent survival to standard PD, leading to an increasing acceptance of vascular resection for curative intent. For example, in 1994, Allema et al. published a series of 20 SMV/PV resections, showing no significant differences in survival in comparison to standard PD and confirmed both the feasibility of the procedure and the ability to obtain R0 resections with vascular resection [11]. Fuhrman et al. confirmed the findings, concluding that vascular resection is a safe and effective means which attains complete resection in cases of tumor adherence to the SMV or SMV/PV confluence [12]. Subsequently, other studies supported the notion that appropriately selected patients could undergo vascular resection to achieve survival outcomes similar to patients undergoing standard PD and superior to outcomes of locally advanced disease treated nonoperatively [13, 14]. In 2004, a study from MD Anderson reviewed all patients who underwent PD from 1990 to 2002 to examine the effect of vascular resection on margin status and survival in PDAC [14]. Of 291 patients who underwent PD for PDAC, 181 had a standard PD and 110 had PD with vascular resection. Median survival was 26.5 months in the standard PD group and 23.4 months in the group that required VR (P*= *0.18). Regardless of these promising findings with vascular resection, it is important to keep in mind that the extent of venous involvement also has a direct relationship to operability and to final margin status [15]. As tumors encroach on the left side of the SMV/PV, they increasingly also encroach on the SMA. Lu et al. reported that tumor involvement of greater than half the circumference was highly specific for unresectable disease [15]. This study by Lu et al. further elucidates the importance of our study which examines the association of both venous and arterial involvement on preoperative imaging to assess whether either can predict positive vascular margins. The importance of preoperatively identifying a positive arterial margin is further evident in a study by Leach at al. They reviewed 75 consecutive patients who underwent a PD; 44 without venous resection; and 31 with en bloc resection of the SMV/PV confluence. There were no perioperative deaths in either group; late (more than 6 months) occlusion of the reconstructed SMV/PV confluence contributed to the death of two patients. Median survival in the 31 patients who required venous resection at the time of PD was 22 months, and for the 44 control patients was 20 months (P = 0.25). They concluded that patients with PDAC of the head of the pancreas who require venous resection during PD for isolated tumor extension to the SMV or SMV/PV confluence (in the absence of tumor extension to the SMA or CA) have a duration of survival no different from that of patients who undergo standard PD [13].

Recent studies have also shown that patients with vascular tumor invasion, who undergo concurrent vascular resection, can achieve long-term survival rates equivalent to those without vascular involvement requiring PD alone. Flis et al. looked at 133 patients who underwent a PD for PDAC, 16.5% of which had a PV/SMV resection [16]. There were no significant differences in postoperative morbidity, mortality, or grades of complication between groups of patients with or without a PVR. Median survival time in months was in a group with vein resection 16.13 months and in a group without vein resection 15.17 months [16]. Five-year survival in the group without vein resection was 19.5%. Comparison of survival curves showed equal hazard rates with log-rank P = 0.090 [16].

Our study suggests that the Ishikawa classification system can predict a positive venous and arterial margin. Ishikawa grading predicted both positive SMA and SMV margins. In particular, Ishikawa grade was strongly associated with a positive SMA margin, and for stages I-IV, a positive SMA margin was seen in 14%, 44%, 53%, and 58%, respectively (p<0.001). However, for arterial grading, there was no association; for arterial grades i-iii, a positive SMA margin was seen in 20%, 40%, and 40%, respectively (p=0.06). Therefore, the Ishikawa classification system was more predicative of arterial involvement. Higher Ishikawa grading was also associated with an increased positive SMV margin, 5%, 26%, 33%, and 33%, for stages I-IV, respectively (p<0.001). Preoperative arterial grading was not predictive of a positive SMV margin (p=0.63). On logistic regression for any positive margin with both venous and arterial staging, only venous staging remained statistically significant in predicting a positive margin.

This study has many strengths and potential limitations. The limitations include its retrospective nature and small Ishikawa types IV and V sample size. Additionally, a weakness of the Ishikawa classification system is that it does not take into consideration CA 19-9 levels, which in our study showed an independent association with overall survival. Furthermore, imaging quality in the 1990s was far inferior to present day imaging; however, the majority of our patients had high-quality abdomen CT scans or angiograms performed to better evaluate vasculature prior to surgery. Another strength is that imaging was reviewed by expert abdominal radiologists. Additionally, the pathology was reviewed by expert pancreatic pathologists using a standard method for over 20 years.

To our knowledge, this is the first study that has compared preoperative arterial and venous staging criteria to assess whether either can predict positive vascular margins. We were able to show that Ishikawa staging in addition to predicting positive venous margins was also more predicative of arterial involvement. This is critical as combined vein resection with PD can only be justified when an R0 resection can be achieved and when there is no direct invasion of the resected vein. If these two requirements cannot be met, then it is difficult to justify a potentially more morbid procedure. Therefore, there is clearly a need for more accurate staging of the interface between tumor and mesenteric vessels to help predict margin status, particularly in the current era of neoadjuvant therapy for BR PDAC.

## 5. Conclusion

This is one of the first studies to show that the use of diagnostic imaging in predicting positive margins is more accurate when using a venous grading system as opposed to an arterial grading system. With a more standard approach of designating degree of vein involvement and improved preoperative imaging, further studies will need to be done to substantiate these findings.

## Figures and Tables

**Table 1:** Patient demographics and characteristics.

	N	%
**Gender**		
Female	76	55.5
Male	61	44.5

**Age**		
38-49	7	5.1
50-59	17	12.4
60-69	42	30.7
70-79	53	38.7
80-84	18	13.1

**Ishikawa staging**		
I: normal portal vein confluence	84	61.3
II: smooth shift	23	16.8
III: unilateral narrowing	15	10.9
IV: bilateral narrowing	12	8.8
Unknown	3	2.2

**Arterial staging**		
i: clean	71	51.8
ii: dirty fat	15	10.9
iii: abutment	10	7.3
Unknown	41	29.9

**Operation**		
Distal Pancreatectomy	1	0.7
Total Pancreatectomy	1	0.7
Total Pancreatectomy, SMV recon	1	0.7
Pancreatoduodenectomy	116	84.7
Pancreatoduodenectomy, HA recon	2	1.5
Pancreatoduodenectomy, PV recon	4	2.9
Pancreatoduodenectomy, SMA recon	1	0.7
Pancreatoduodenectomy, SMV recon	11	8.0

**Adjuvant Therapy**		
No	24	17.5
Yes	109	79.6
Unknown	4	2.9

**Positive lymph nodes**		
No	36	26.3
Yes	100	73.0
Unknown	1	0.7

**CA 19-9**		
<50	39	28.5
50-250	41	29.9
>250	56	40.9
Unknown	1	0.7

**(a):** 

	*N*	Any Positive Margin			SMA	SMV		
Ishikawa Classification		*N*	%	*P*-value	*N*	*N*	%	*P*-value
I: normal portal vein confl	84	19	22.6	0.0001	12	4	4.8	0.0001
II: smooth shift	23	13	56.5		10	6	26.1	
III: unilateral narrowing	15	11	73.3		8	5	33.3	
IV: bilateral narrowing	12	8	66.7		7	4	33.3	

**(b):** 

	*N*	Any Positive Margin			SMA			SMV		
Arterial Classification		*N*	%	*P*-value	*N*	%	*P*-value	*N*	%	*P*-value
i: clean	71	20	28.2	<0.0001	14	19.7	0.062	7	9.9	0.63
ii: dirty fat	15	10	66.7		6	40.0		3	20.0	
iii: abutment	10	5	50.0		4	40.0		1	10.0	

**Table 3:** Multivariate analysis of vascular classification and margin status.

	Any Positive Margin		Positive SMA Margin		Positive SMV Margin	
	*N* = 33		*N* = 23		*N* = 10	
	OR	*P*-value	OR	*P*-value	OR	*P*-value
**Ishikawa Classification**						
I	1 (ref)		1 (ref)		1 (ref)	
II-IV	4.3	0.0023	4.4	0.0036	3.9	0.048

**Arterial Classification**						
i	1 (ref)		1 (ref)		1 (ref)	
ii-iii	4.2	0.0046	3.4	0.020	1.4	0.68

**Combined**		0.2081		0.8		0.79
Both Clean	1 (ref)		1 (ref)		1 (ref)	
Both Involved	9.2	0.0084	6.1	0.0243	3.1	0.0084

**Table 4:** Multivariate analysis of vascular and nonvascular variables on margin status.

	Any Positive Margin		Positive SMA Margin	
	*N* = 33		*N* = 23	
	OR	*P*-value	OR	*P*-value
**Vascular classification**		0.0083		0.03
Both Clean	1 (ref)		1 (ref)	
Vein un-involved, Artery involved	0.9	0.93	2.5	0.33
Artery un-involved, Vein involved	1.7	0.43	3.6	0.07
Both Involved	10.2	0.0007	6.3	0.0044

**Positive lymph nodes**				
No	1 (ref)		1 (ref)	
Yes	3.0	0.069	1.0	0.97

**CA 19-9 level**		0.93		0.28
<50	1 (ref)		1 (ref)	
50-250	0.8	0.76	0.8	0.72
>250	1	0.98	2.0	0.26

**Table 5:** Overall survival estimates at 12, 24, and 36 months by Ishikawa and arterial staging groups.

	*N*	*P*-value	12 mos	LCL	UCL	24 mos	LCL	UCL	36 mos	LCL	UCL
**Ishikawa**											
I	84	0.0056	82.9%	72.8%	89.5%	50.8%	39.3%	61.3%	29.8%	19.9%	40.4%
II-IV	50		62.0%	47.1%	73.8%	32.0%	19.7%	45.0%	14.0%	6.2%	25.0%

**Arterial classification**											
i	71	0.0422	82.6%	71.5%	89.7%	49.5%	37.0%	60.8%	28.7%	18.3%	40.0%
ii-iii	25		64.0%	42.2%	79.4%	36.0%	18.2%	54.2%	8.0%	1.4%	22.5%

**Combined**											
Both clean	57	0.2981	83.7%	71.0%	91.2%	49.2%	35.0%	61.8%	28.7%	17.0%	41.5%
One involved	20		85.0%	60.4%	94.9%	50.0%	27.1%	69.2%	20.0%	6.2%	39.3%
Both involved	16		50.0%	24.5%	71.0%	37.5%	15.4%	59.8%	12.5%	2.1%	32.8%

LCL: lower confidence level; UCL: upper confidence level.

## Data Availability

The data in the discussion section are taken from the articles that are included in the references section. Our data is stored on a locked Fox Chase Cancer Center computer.
